# Physical Activity, Dietary Inflammatory Potential, and Depressive Symptoms Among U.S. Adults: The Potential Contribution of Oral Microbial Dysbiosis

**DOI:** 10.3390/healthcare14172795

**Published:** 2026-09-01

**Authors:** Leizi Min, Yicong Cui, Xindong Ma, Longteng Cui

**Affiliations:** 1 Division of Sports Science and Physical Education, Tsinghua University, Beijing 100084, China; minlz23@mails.tsinghua.edu.cn (L.M.); cuiyicong@ecust.edu.cn (Y.C.);; 2 IDG/McGovern Institute for Brain Research, Tsinghua University, Beijing 100082, China

**Keywords:** lifestyle behaviors, health promotion, physical activity, dietary inflammatory potential, depressive symptoms, oral microbial dysbiosis, mental well-being

## Abstract

**Background/Objectives**: Physical activity and dietary inflammatory potential are modifiable correlates of depressive symptoms. We examined whether oral microbial dysbiosis—an imbalance in oral microbial community structure—was associated with an integrated lifestyle profile and depressive symptoms in U.S. adults. **Methods**: We analyzed 6519 adults aged 20–69 years from NHANES 2009–2012. The Integrated Lifestyle Score combined standardized Dietary Inflammatory Index and leisure-time physical activity; higher scores indicated higher dietary inflammatory potential and lower physical activity. Oral dysbiosis was assessed using a study-specific genus-level log-ratio index. Survey-weighted regression models were fitted, and indirect associations were evaluated using 5000 bootstrap resamples. **Results**: A less favorable lifestyle profile, characterized by higher dietary inflammatory potential and lower leisure-time physical activity, was associated with higher PHQ-9 scores (β = 0.314, 95% CI 0.113–0.515, *p* = 0.005), greater odds of clinically relevant depressive symptoms (PHQ-9 ≥ 10; OR = 1.355, 95% CI 1.159–1.584, *p* < 0.001), and greater oral dysbiosis (β = 0.191, 95% CI 0.050–0.331, *p* = 0.011). Greater oral dysbiosis was associated with higher PHQ-9 scores (β = 0.101, 95% CI 0.059–0.144, *p* < 0.001) and greater odds of clinically relevant depressive symptoms (OR = 1.069, 95% CI 1.035–1.105, *p* < 0.001). The indirect association for continuous PHQ-9 was 0.0188 (bootstrap 95% CI 0.0033–0.0402), representing approximately 6.0% of the total association. **Conclusions**: A less favorable lifestyle profile was associated with greater depressive symptom burden and oral microbial imbalance. Oral dysbiosis statistically accounted for a small proportion of this association. Given the cross-sectional design and study-specific indices, these findings should be interpreted as exploratory rather than causal.

## 1. Introduction

Depressive disorders remain a major contributor to disability worldwide, underscoring the need for prevention strategies that complement clinical treatment [1,2]. Among potentially modifiable correlates, lifestyle behaviors are especially relevant because they are common, amenable to intervention, and tend to cluster rather than occur in isolation [3]. Clarifying how such behavioral patterns relate to depressive symptoms may therefore inform population-level prevention while also helping to identify biological processes that accompany these associations.

Physical activity and diet are two lifestyle dimensions with particularly consistent links to depression. Higher physical activity has been associated with lower depressive symptom burden and lower risk of depression [4], whereas diets with greater inflammatory potential, quantified using the Dietary Inflammatory Index (DII), have been associated with higher depression risk [5]. These measures capture different aspects of behavior: physical activity reflects movement-related energy expenditure, whereas the DII summarizes the inflammatory potential of dietary intake [6]. They should therefore be viewed as complementary lifestyle dimensions rather than as a comprehensive measure of lifestyle. How their joint pattern relates to biological variation relevant to depressive symptoms remains incompletely understood.

Inflammatory and neuroimmune processes are increasingly implicated in major depressive disorder [7]. In parallel, microbial ecosystems have emerged as potential interfaces between environmental exposures and host physiology. Although most microbiota–mental health research has focused on the gut microbiome [8], the oral microbiome is a distinct and highly structured microbial ecosystem that is continuously exposed to dietary, behavioral, and host-related influences [9,10]. Oral dysbiosis is best understood as a disturbance in community organization and ecological balance rather than the presence or absence of a single microorganism [9,10,11]. This ecological perspective makes the oral microbiome a plausible population-level correlate of lifestyle-related exposures.

Oral microbial alterations have been linked to local and systemic inflammatory processes and to a range of extra-oral health conditions [11,12,13]. More recently, population-based studies have reported associations between oral microbiome characteristics and depressive symptoms [14,15], and a 2025 systematic review suggested that an oral dysbiosis–depression relationship is plausible but remains heterogeneous across microbial measures and study populations [16]. Importantly, most previous investigations have examined behavioral exposures and oral microbial characteristics separately. It therefore remains unclear whether physical activity and dietary inflammatory potential, considered jointly, are associated with oral microbial imbalance and whether oral dysbiosis statistically accounts for part of their association with depressive symptoms.

Using nationally representative data from the National Health and Nutrition Examination Survey (NHANES) 2009–2012, we examined the relationships among an integrated lifestyle profile, oral microbial dysbiosis, and depressive symptoms in U.S. adults. Specifically, we evaluated (1) the association of the integrated lifestyle profile with depressive symptoms, (2) its association with oral microbial dysbiosis, and (3) the extent to which oral dysbiosis statistically accounted for part of the observed lifestyle–depressive symptom association. Because the exposure, mediator, and outcome were measured cross-sectionally, the mediation analysis was treated as an exploratory decomposition of statistical associations rather than evidence of a temporal or causal pathway.

## 2. Materials and Methods

### 2.1. Study Design and Population

This cross-sectional study used data from the 2009–2010 and 2011–2012 cycles of NHANES, conducted by the National Center for Health Statistics (NCHS). NHANES uses a complex, stratified, multistage probability sampling design to obtain health and nutritional information representative of the civilian, noninstitutionalized U.S. population [17]. These two cycles were selected because they jointly provide the measures required for the present analysis, including depressive symptoms, physical activity, Day 1 dietary intake, and oral microbiome data [18,19].

**Figure 1:**
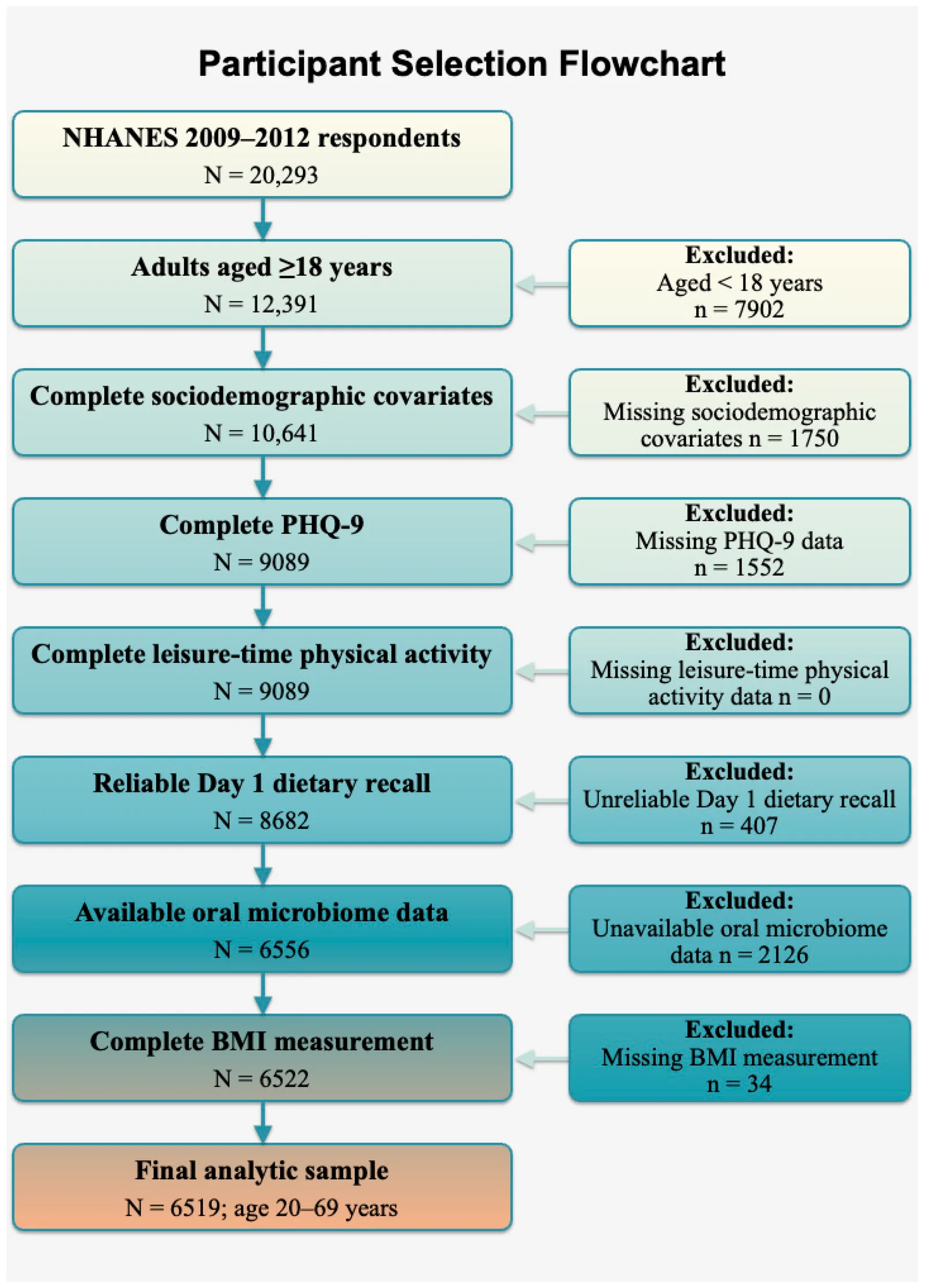
Participant selection flowchart for the NHANES 2009–2012 analytic sample.

Adults aged ≥18 years were initially eligible. Participants were then required to have complete information on depressive symptoms, physical activity, dietary intake, oral microbiome measures, and prespecified covariates. After these sequential complete-case restrictions, the final analytic sample comprised adults aged 20–69 years. A sequential selection procedure was used so that each exclusion was counted only once; the participant-selection process is presented in Figure 1.

Because this was a secondary analysis of fixed NHANES cycles, no a priori sample-size calculation was performed. All eligible participants meeting the prespecified complete-case requirements were included in the analytic sample. Characteristics of included and nonincluded adults were compared descriptively to assess potential selection differences (Table S1, Panel A).

NHANES protocols were reviewed and approved by the NCHS Research Ethics Review Board. The 2009–2010 cycle was conducted under a continuation of Protocol #2005-06, and the 2011–2012 cycle was approved under Protocol #2011-17. All participants provided written informed consent.

### 2.2. Assessment of Depressive Symptoms

Depressive symptoms were assessed using the Patient Health Questionnaire-9 (PHQ-9), a validated nine-item measure of depressive symptom severity [20]. Participants reported how often they had experienced each symptom during the preceding two weeks. Each item was scored from 0 to 3, yielding a total score from 0 to 27, with higher scores indicating greater symptom severity. The PHQ-9 total score was treated as the primary continuous outcome. In complementary analyses, clinically relevant depressive symptoms were defined as PHQ-9 ≥ 10 and analyzed as a binary outcome [20].

### 2.3. Assessment of Lifestyle Behaviors

#### 2.3.1. Physical Activity

Physical activity was assessed using the NHANES Physical Activity Questionnaire. The present analysis focused on leisure-time moderate- and vigorous-intensity activity. Vigorous leisure-time activity was derived from PAQ650, PAQ655, and PAD660, and moderate leisure-time activity from PAQ665, PAQ670, and PAD675. Consistent with the Global Physical Activity Questionnaire scoring convention, vigorous- and moderate-intensity activities were assigned values of 8 and 4 metabolic equivalents (METs), respectively [21]. Weekly MET-minutes were calculated as MET value × days/week × minutes/session for each intensity and then summed to obtain total leisure-time MET-min/week. Transportation and occupational activity were not included.

Physical activity was modeled continuously. No categorical threshold was used to define low physical activity in the primary exposure, and higher MET-min/week values indicated greater leisure-time physical activity.

#### 2.3.2. Dietary Inflammatory Potential

Dietary inflammatory potential was assessed using the Dietary Inflammatory Index (DII), a literature-derived score developed to quantify the inflammatory potential of dietary intake [6]. Dietary intake was derived from the first 24 h dietary recall (Day 1; NHANES DR1TOT files). Recalls with DR1DRSTZ ≠ 1 or total energy intake <500 or >5000 kcal/day were excluded according to the prespecified analytic rule. DII scores were calculated from 26 dietary parameters available in the analytic dataset using the global reference standardization and inflammatory-effect scoring procedure described by Shivappa et al. [6]. The 26 parameters are listed in the Supplementary Methods.

Higher DII values indicate a more pro-inflammatory dietary profile, whereas lower values, including negative values, indicate a relatively more anti-inflammatory profile. Distributional characteristics of the DII in the analytic sample, including its observed range, are reported in Table S1, Panel B.

### 2.4. Construction of the Integrated Lifestyle Score

Physical activity and dietary inflammatory potential were combined into a single continuous exposure to summarize the two prespecified lifestyle dimensions examined in this study. The composite was developed specifically for the present analysis and was intended as an exploratory integrated exposure measure rather than a validated clinical, diagnostic, or prediction score. It did not include other lifestyle domains such as sleep, smoking, or alcohol use.

DII and MET-min/week were standardized using Z-score transformations. To derive sample-specific weights, a survey-weighted multivariable linear regression model was fitted with continuous PHQ-9 score as the outcome and Z(DII) and Z(MET) entered simultaneously, with adjustment for age, sex, race/ethnicity, educational attainment, poverty–income ratio (PIR), and marital status. The absolute standardized coefficients for DII (β = 0.2756) and physical activity (β = −0.2384) were divided by their sum, yielding normalized weights of 0.5362 and 0.4638, respectively. Because higher DII reflects a less favorable dietary profile whereas higher physical activity reflects a more favorable behavioral profile, the physical-activity component entered the composite in the opposite direction:

Integrated Lifestyle Score = 0.5362 × Z(DII) − 0.4638 × Z(MET-min/week)

Higher Integrated Lifestyle Scores therefore represented less favorable lifestyle profiles characterized by greater dietary inflammatory potential and lower physical activity. The score was analyzed continuously in the primary regression models. In the analytic sample, the score ranged from −5.76 to 1.40. For descriptive comparisons in Table 1 only, participants were divided at the sample median (0.112) into a more favorable lifestyle profile group (score ≤ median) and a less favorable lifestyle profile group (score > median). This dichotomization was not used to define the primary exposure in inferential models.

Because the weighting was derived within the same analytic sample and has not been externally validated, the composite was interpreted as sample-specific. Component-level robustness analyses examined standardized physical activity and DII separately and jointly, as well as evaluating their associations with the Oral Dysbiosis Index (Table S2). These analyses were considered robustness analyses rather than validation of the score.

### 2.5. Oral Microbiome Assessment

Oral microbiome data were obtained from oral rinse specimens collected during the 2009–2010 and 2011–2012 NHANES cycles [18,19]. The V4 region of the bacterial 16S ribosomal RNA gene was amplified and sequenced using the Illumina HiSeq 2500 platform. Forward reads were processed using DADA2 to infer amplicon sequence variants (ASVs), and taxonomic assignment was performed against the SILVA version 123 reference database [18,19]. Genus-level relative abundance data were used in the present analyses.

### 2.6. Construction of the Oral Microbial Dysbiosis Index

A study-specific genus-level log-ratio index was constructed to summarize the relative balance of six prespecified oral bacterial genera. Its conceptual basis was ecological: oral dysbiosis reflects shifts in community organization and ecological balance rather than the presence of a single pathogen [9,10,11,22]. Because ecological roles can vary by species, strain, site, and host context, the six genera were described neutrally as numerator and denominator components rather than as universally pathogenic or healthy taxa.

The numerator comprised the relative abundances of *Lactobacillus*, *Scardovia*, and *Veillonella*, whereas the denominator comprised *Neisseria*, *Aggregatibacter* (annotated as *Actinobacillus* in the NHANES SILVA v123 taxonomy file), and *Capnocytophaga*. The index was calculated as:

Oral Dysbiosis Index = log_2_[(Σ numerator genera + ε)/(Σ denominator genera + ε)]

The pseudocount ε was defined in the analysis code as one-half of the smallest nonzero genus-level relative abundance observed in the complete genus-level matrix (ε = 1.48 × 10^−6^), allowing the log-ratio to be calculated in the presence of zero abundances. Higher index values indicate a greater relative predominance of the numerator genera over the denominator genera. The index was analyzed primarily as a continuous variable.

This exact six-genus composite is investigator-defined and has not undergone independent validation, reproducibility testing, or evaluation of diagnostic or predictive performance. It was therefore treated as an exploratory ecological index. Internal robustness was assessed using leave-one-genus-out analyses in which the index was recalculated after sequential omission of each genus (Table S3, Panel B); these analyses were not considered a substitute for external validation.

### 2.7. Covariates

Covariates were prespecified on the basis of prior epidemiological evidence and their plausible associations with lifestyle behaviors, depressive symptoms, or oral microbial composition. They included age, sex, race/ethnicity, educational attainment, PIR, marital status, body mass index (BMI), smoking status, and alcohol consumption. Model 2 adjusted for age, sex, race/ethnicity, educational attainment, PIR, and marital status. Model 3 additionally adjusted for BMI category, smoking status, and alcohol consumption.

### 2.8. Statistical Analysis

All survey-weighted analyses used the NHANES Day 1 dietary sample weight (WTDRD1), because the Dietary Inflammatory Index was derived from the Day 1 24 h dietary recall and this dietary recall represents the smallest applicable subsample for the variables used in the analysis. Because two 2-year cycles (2009–2010 and 2011–2012) were combined, a 4-year analysis weight was constructed by dividing each participant’s 2-year Day 1 dietary weight by two (WT4YR = WTDRD1/2), in accordance with NCHS analytic guidance [17]. Sampling strata (SDMVSTRA) and primary sampling units (SDMVPSU) were incorporated using Taylor-series linearization.

Continuous-variable distributions were evaluated using histograms, Q–Q plots, skewness, and descriptive statistics. For descriptive presentation, variables with approximately symmetric distributions (|skewness| < 0.5) were summarized using survey-weighted means and standard deviations, whereas clearly skewed variables were summarized using survey-weighted medians and interquartile ranges. Categorical variables were summarized using survey-weighted percentages. Distributional diagnostics are summarized in Table S1, Panel B.

For Table 1 comparisons, approximately symmetric continuous variables were compared using survey-weighted t tests, skewed continuous variables using survey-weighted rank tests, and categorical variables using Rao–Scott adjusted chi-square tests. The descriptive lifestyle groups were defined using the sample median of the Integrated Lifestyle Score, as described above.

Survey-weighted linear regression was used when PHQ-9 score or the Oral Dysbiosis Index was analyzed as a continuous outcome. Survey-weighted logistic regression was used for clinically relevant depressive symptoms defined as PHQ-9 ≥ 10. Three sequential models were fitted: Model 1 was unadjusted; Model 2 adjusted for age, sex, race/ethnicity, educational attainment, PIR, and marital status; and Model 3 additionally adjusted for BMI category, smoking status, and alcohol consumption.

Multicollinearity was assessed for the fully adjusted predictor set using variance inflation factors (VIFs) for single-degree-of-freedom predictors and generalized variance inflation factors (GVIFs) for multi-level categorical predictors. GVIFs were dimension-adjusted using GVIF^(1/(2 × df)) in accordance with Fox and Monette [23]. A VIF-like value < 5 was used as the prespecified criterion for acceptable collinearity. Detailed diagnostics are reported in Table S3, Panel A.

Component-level robustness analyses examined Z(PA) and Z(DII) separately in relation to continuous PHQ-9 score and binary clinically relevant depressive symptoms using the same sequential adjustment strategy; fully adjusted joint models containing both components were also fitted. The individual lifestyle components were additionally examined in relation to the Oral Dysbiosis Index (Table S2).

The indirect association through oral microbial dysbiosis was evaluated using nonparametric bootstrap resampling with 5000 iterations, following established resampling approaches for indirect-effect estimation [24]. For each outcome, path a represented the association between the Integrated Lifestyle Score and the Oral Dysbiosis Index; path b represented the association between the Oral Dysbiosis Index and the outcome conditional on the lifestyle score; path c represented the total association; and path c′ represented the direct association conditional on the mediator. The indirect association was calculated as a × b, and percentile-based 95% bootstrap confidence intervals were obtained. An indirect association was considered statistically supported when the bootstrap confidence interval did not include zero.

The bootstrap used individual-level nonparametric case resampling rather than a design-based replicate-weight bootstrap. Within each resample, survey-weighted regression models were refitted using the NHANES design variables carried into the resampled dataset. Accordingly, the bootstrap analysis was interpreted as an exploratory estimate of the indirect statistical association and was not described as a fully design-based survey bootstrap. Because exposure, mediator, and outcome were measured contemporaneously, no temporal or causal mediation claim was made.

Additional robustness analyses included leave-one-genus-out recalculation of the oral microbial index (Table S3, Panel B). Exploratory stratified analyses for the continuous PHQ-9 outcome were also conducted by smoking status and alcohol consumption (Table S4). Within smoking-status strata, smoking status was omitted from the adjustment set; within alcohol-consumption strata, alcohol consumption was omitted. No subgroup-specific bootstrap inference or formal between-stratum comparisons were performed; therefore, the stratified estimates were presented descriptively.

All statistical tests were two-sided, and *p* < 0.05 was considered statistically significant. Analyses were performed using R version 4.4.2 (R Core Team, Vienna, Austria), including the survey package for design-based regression and the boot package for nonparametric resampling.

## 3. Results

### 3.1. Participant Selection and Characteristics

Of 20,293 NHANES participants from the 2009–2010 and 2011–2012 cycles, 12,391 were aged ≥18 years and were initially eligible. Sequential complete-case restrictions for sociodemographic variables, PHQ-9, physical activity, Day 1 dietary recall, oral microbiome data, BMI, and smoking status yielded a final analytic sample of 6519 participants aged 20–69 years (Figure 1). Among these participants, 659 met the PHQ-9 ≥ 10 criterion on an unweighted-count basis.

Survey-weighted participant characteristics are summarized in Table 1. Participants were classified for descriptive purposes as having a more favorable or less favorable lifestyle profile using the sample median of the Integrated Lifestyle Score (cutoff = 0.112). The less favorable lifestyle group had a higher DII, substantially lower leisure-time physical activity, a higher Oral Dysbiosis Index, and greater depressive symptom burden than the more favorable group. Because several continuous variables were markedly skewed, BMI, physical activity, the Integrated Lifestyle Score, Oral Dysbiosis Index, and PHQ-9 are presented as medians with interquartile ranges, whereas age and DII are summarized using means and standard deviations.

In supplementary comparisons, included participants were younger and had slightly higher BMI than nonincluded adults, whereas sex and non-Hispanic White proportions were broadly similar (Table S1, Panel A). Distributional characteristics of continuous variables are provided in Table S1, Panel B.

### 3.2. Integrated Lifestyle Score and Depressive Symptoms

Higher Integrated Lifestyle Scores were associated with greater depressive symptom burden across sequential survey-weighted models (Table 2). For continuous PHQ-9, the association attenuated after covariate adjustment but remained statistically significant in Model 3 (β = 0.314, 95% CI 0.113–0.515, *p* = 0.005). For clinically relevant depressive symptoms (PHQ-9 ≥ 10), each one-unit increase in the score was associated with 35% higher odds in the fully adjusted model (OR = 1.355, 95% CI 1.159–1.584, *p* < 0.001).

Component-level robustness analyses were consistent with the composite score (Table S2, Panels A–B). For continuous PHQ-9, higher standardized physical activity remained inversely associated and higher standardized DII remained positively associated after full adjustment; both associations also remained statistically significant when the two components were entered jointly. For the binary PHQ-9 ≥ 10 outcome, DII remained positively associated in the fully adjusted and joint models, whereas the inverse association with physical activity was attenuated and did not reach statistical significance in those models. These findings support robustness of the direction of the composite association but do not constitute validation of the Integrated Lifestyle Score.

### 3.3. Integrated Lifestyle Score and Oral Microbial Dysbiosis

The Integrated Lifestyle Score was positively associated with the study-specific Oral Dysbiosis Index (Table 3). The association remained statistically significant after full covariate adjustment (β = 0.191, 95% CI 0.050–0.331, *p* = 0.011), indicating that less favorable lifestyle profiles were associated with greater relative predominance of the numerator genera over the denominator genera used to construct the index.

The Oral Dysbiosis Index was analyzed continuously in the primary analysis. Component-level analyses showed that higher DII was positively associated with the index and greater physical activity was inversely associated with the index after full adjustment (DII: β = 0.077, *p* = 0.039; physical activity per 1000 MET-min/week: β = −0.040, *p* = 0.031; Table S2, Panel C).

### 3.4. Oral Microbial Dysbiosis and Depressive Symptoms

Higher Oral Dysbiosis Index values were associated with greater depressive symptom burden (Table 4). In the fully adjusted linear model, each one-unit increase in the index was associated with a 0.101-point higher PHQ-9 score (β = 0.101, 95% CI 0.059–0.144, *p* < 0.001). In the corresponding logistic model, each one-unit increase was associated with 6.9% higher odds of clinically relevant depressive symptoms (OR = 1.069, 95% CI 1.035–1.105, *p* < 0.001).

Leave-one-genus-out analyses yielded positive associations with PHQ-9 regardless of which of the six genera was removed, and the recalculated indices remained correlated with the full index (r = 0.702–0.996), suggesting that the observed association was not driven by a single genus (Table S3, Panel B).

### 3.5. Exploratory Indirect-Association Analysis

Bootstrap analysis supported a statistically detectable indirect association through oral microbial dysbiosis. For continuous PHQ-9, path a (Integrated Lifestyle Score → Oral Dysbiosis Index) was 0.1908 and path b (Oral Dysbiosis Index → PHQ-9, conditional on the lifestyle score and covariates) was 0.0986. The total association was 0.3139, and the estimated indirect association was 0.0188 (5000-resample bootstrap 95% CI 0.0033–0.0402), corresponding to approximately 6.0% of the total statistical association. After conditioning on the Oral Dysbiosis Index and covariates, the direct association remained statistically significant (c′ = 0.2951, SE = 0.0946, 95% CI 0.0923–0.4980, *p* = 0.008).

**Figure 2:**
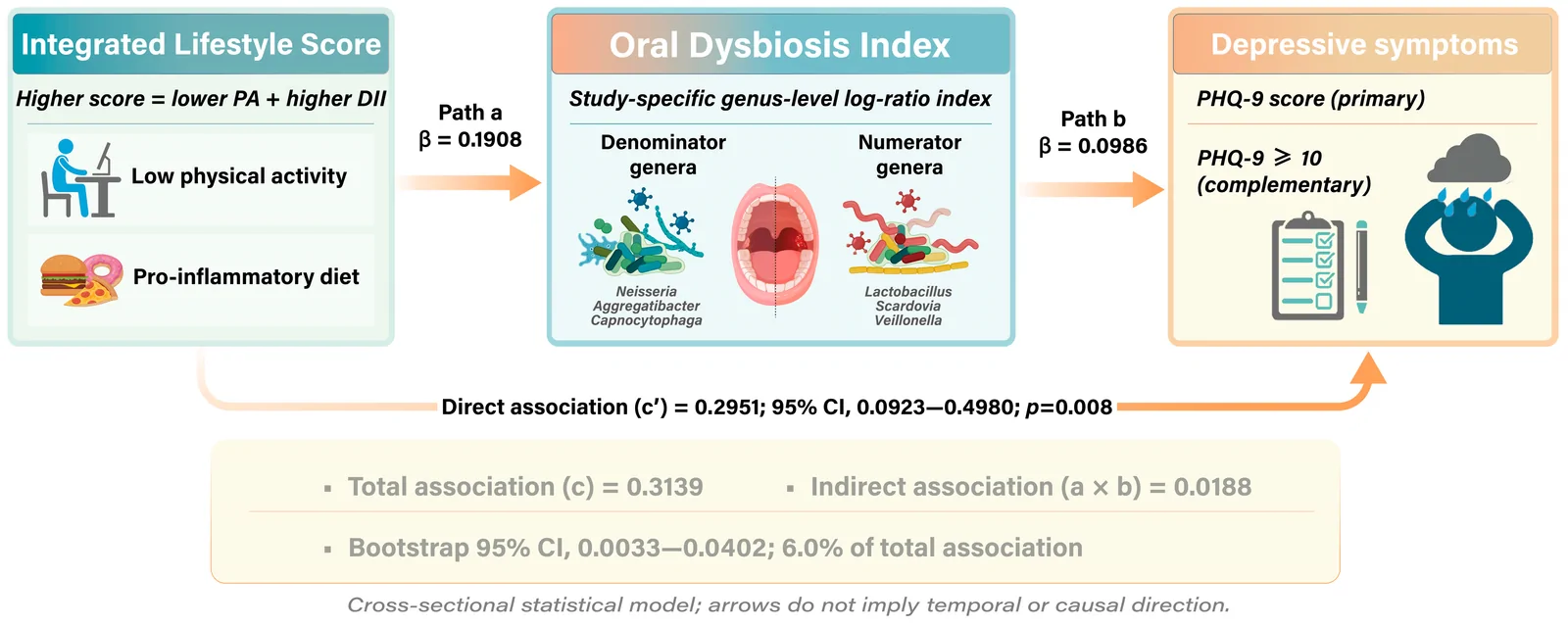
Exploratory indirect-association model linking the Integrated Lifestyle Score, Oral Dysbiosis Index, and depressive symptoms. Path coefficients are shown for the continuous PHQ-9 outcome. The indirect association was estimated using 5000 bootstrap resamples. Arrows represent the prespecified statistical model and do not imply temporal or causal direction.

For the binary PHQ-9 ≥ 10 outcome, the estimated indirect association was 0.0125 (bootstrap 95% CI 0.0022–0.0272), corresponding to approximately 4.1% of the total association. These findings were interpreted as an exploratory decomposition of cross-sectional associations rather than evidence of temporal or causal mediation. These findings were interpreted as an exploratory decomposition of cross-sectional associations rather than evidence of temporal or causal mediation. The corresponding exploratory indirect-association model is presented in Figure 2.

### 3.6. Robustness and Stratified Analyses

No substantial multicollinearity was identified in the fully adjusted covariate set; the maximum VIF-like value was 1.40 (Table S3, Panel A). Exploratory stratified analyses showed positive indirect-association point estimates across all smoking-status strata. Alcohol-stratified estimates were more heterogeneous, including a small negative estimate in the high-alcohol stratum (Table S4). Because no subgroup-specific bootstrap inference or formal between-stratum comparison was performed, no inferential conclusions were drawn from these exploratory analyses.

## 4. Discussion

### 4.1. Principal Findings

In this survey-weighted analysis of 6519 U.S. adults, a less favorable integrated lifestyle profile—characterized by greater dietary inflammatory potential and lower leisure-time physical activity—was associated with greater depressive symptom burden and with a higher study-specific Oral Dysbiosis Index. The oral index was, in turn, positively associated with both continuous PHQ-9 scores and clinically relevant depressive symptoms. These findings are directionally consistent with prior evidence linking physical activity and dietary inflammatory potential with depression [3,4,5] and with recent population-based studies relating oral microbial characteristics to depressive symptoms [14,15,16].

The bootstrap analysis identified a small but statistically detectable indirect association through oral microbial dysbiosis. For continuous PHQ-9, the indirect association was 0.0188 (95% CI 0.0033–0.0402), corresponding to approximately 6.0% of the total statistical association; the direct association remained significant after conditioning on the oral index (c′ = 0.2951, 95% CI 0.0923–0.4980, *p* = 0.008). The corresponding indirect proportion for PHQ-9 ≥ 10 was approximately 4.1%. These estimates suggest that the oral microbial measure captures only a modest part of the observed lifestyle–depressive symptom relationship and should not be interpreted as evidence of a dominant biological pathway.

The component-level analyses strengthen the directional interpretation of the composite. For continuous PHQ-9, higher physical activity and lower DII remained significantly associated with lower depressive symptom burden after full adjustment and when both components were entered jointly. For the binary PHQ-9 ≥ 10 outcome, DII remained positively associated in the fully adjusted and joint models, whereas the inverse association with physical activity was attenuated and did not reach statistical significance. Both DII and physical activity were also associated with the Oral Dysbiosis Index in the expected directions after full adjustment. These findings support robustness of the composite association, but they do not demonstrate biological synergy between physical activity and diet.

### 4.2. Integrated Lifestyle Profile and Depressive Symptoms

The association between a less favorable lifestyle profile and greater depressive symptom burden accords with a broad literature on modifiable behavioral correlates of depression [3,4,5]. Physical activity may influence mood through neuroplastic, neuroendocrine, sleep-related, and inflammatory pathways, and randomized evidence supports exercise as an effective treatment component for depression, although effect sizes and certainty vary across exercise modes and study designs [25]. Diet may operate through partly overlapping metabolic and immune pathways; randomized-trial evidence also suggests that Mediterranean-style dietary interventions can reduce depressive symptoms in some adult populations, while heterogeneity across trials remains substantial [26].

The component analyses nevertheless support a differentiated interpretation of the two lifestyle dimensions. For continuous PHQ-9, both standardized physical activity and DII remained statistically significant in the fully adjusted joint model. For PHQ-9 ≥ 10, DII remained significant whereas physical activity was attenuated after full adjustment. The composite score may therefore capture shared and complementary information across the two domains. At the same time, because the 0.5362/0.4638 weights were derived from PHQ-9 associations within the same analytic sample, the score is sample-specific and may be susceptible to optimism. The component analyses show that the direction of the main findings is not solely an artifact of the exact weighting scheme, but they do not constitute internal or external validation.

### 4.3. Integrated Lifestyle Profile and Oral Microbial Dysbiosis

A less favorable integrated lifestyle profile was also associated with a higher Oral Dysbiosis Index after adjustment for sociodemographic characteristics, BMI, smoking, and alcohol consumption. Oral microbial composition is responsive to multiple behavioral and host exposures. Tobacco use has been associated with reproducible shifts in oral microbial communities [27], while a recent systematic review identified diet and nutrient composition as important determinants of oral microbial structure and ecological balance [28]. These observations are relevant because the oral cavity is directly and repeatedly exposed to dietary substrates, whereas physical activity is more likely to influence oral ecology indirectly through systemic metabolic, inflammatory, salivary, or behavioral pathways.

This pattern is also reflected in the component results. Higher DII remained positively associated with the oral index after full adjustment, and greater physical activity remained inversely associated with the index when expressed per 1000 MET-min/week. Thus, both components provided directionally consistent evidence for the lifestyle–oral microbiome association in the fully adjusted analysis. The broader oral–gut microbiome literature further emphasizes that oral and intestinal microbial ecosystems are connected through microbial transmission, host immunity, and shared environmental exposures [29], providing plausible biological context for systemic associations without establishing a specific causal route in this study.

Residual confounding remains possible. Oral microbial composition may vary with age, socioeconomic conditions, smoking, alcohol use, oral-hygiene practices, periodontal disease, dental treatment, medication use, and other host characteristics [9,12,27,28,29]. The present models accounted for several of these factors, but the analytic framework did not contain a complete set of oral-health and treatment variables. The observed lifestyle–oral microbiome association should therefore be interpreted as an adjusted population-level association rather than a direct effect of lifestyle on microbial ecology.

### 4.4. Oral Microbial Dysbiosis, Depressive Symptoms, and the Indirect Association

The positive association between the Oral Dysbiosis Index and depressive symptoms is consistent with an emerging literature connecting the oral microbiome with mental health. Recent reviews describe several potential routes linking oral microbial disturbance with neuropsychiatric outcomes, including systemic inflammation, altered barrier function, immune signaling, and oral–gut–brain communication [30]. A 2025 systematic review and meta-analysis across mental disorders also reported reproducible differences in oral microbial composition, while emphasizing heterogeneity across diagnoses, sampling sites, sequencing methods, and study populations [31]. Together with the depression-specific evidence cited above [14,15,16], these studies support further investigation of the oral microbiome as a correlate of mental health rather than as an established biomarker or therapeutic target.

The study-specific Oral Dysbiosis Index requires particular caution. The six-genus log-ratio is compatible with the general principle that sequencing-derived microbiome data are compositional and are often more appropriately interpreted through relative rather than absolute abundance relationships [32]. However, that statistical rationale does not validate the biological classification of this exact six-genus combination. Leave-one-genus-out analyses showed that the association with PHQ-9 persisted regardless of which genus was omitted, supporting internal robustness, but the index has not been externally validated for reproducibility, discrimination, or prediction.

The indirect-association model should be interpreted with the same restraint. The indirect estimates were statistically detectable but modest, and a substantial direct association remained. More importantly, NHANES is cross-sectional. The assumed ordering of lifestyle profile → oral dysbiosis → depressive symptoms cannot be established temporally, and reverse or reciprocal pathways are plausible. Depressive symptoms may alter appetite, food choice, physical activity, smoking or alcohol use, oral-hygiene practices, and health-care seeking, each of which could subsequently influence the oral microbiome. The mediation analysis is therefore best regarded as a statistical decomposition of contemporaneous associations rather than evidence that oral dysbiosis causally transmits an effect of lifestyle to depression.

### 4.5. Public Health and Research Implications

The results support continued attention to modifiable behaviors, but they do not establish intervention thresholds. Current World Health Organization guidance recommends that adults accumulate 150–300 min/week of moderate-intensity aerobic activity, 75–150 min/week of vigorous-intensity activity, or an equivalent combination [33]. These values provide an external public-health benchmark and should not be interpreted as cut points derived from the present analysis. Similarly, the DII findings are compatible with dietary patterns emphasizing vegetables, fruits, legumes, whole grains, nuts, fish, and unsaturated fats while limiting highly refined and heavily processed foods; Mediterranean-style dietary interventions provide one evidence-based example, although the present cross-sectional analysis cannot establish treatment effects [26].

The component-level oral microbiome results should still be interpreted cautiously. Greater physical activity and lower dietary inflammatory potential were both associated with a more favorable value of the study-specific oral index after full adjustment, but the cross-sectional design does not establish that changing either behavior would modify oral microbial ecology. Physical activity remains relevant to depressive health based on a broader evidence base [4,25,33], and dietary inflammatory potential also showed an independent association with the oral microbial measure in this dataset. Longitudinal and interventional studies are needed to determine whether changes in diet or activity precede changes in oral microbial ecology and whether those microbial changes predict subsequent depressive symptoms.

Future work should also move beyond the two-component lifestyle score. Sleep, oral-hygiene behaviors, dental care, medication exposure, and psychosocial context may materially influence both depressive symptoms and microbial ecology. Social connection is increasingly recognized as a determinant of mental and physical health [34]. Religious and spiritual dimensions have also been associated with depressive trajectories in prospective studies, although effects vary by population, construct, and cultural context [35]. These domains should be incorporated selectively and theoretically rather than simply added to a larger score.

### 4.6. Strengths and Limitations

Several features strengthen this study. The analysis used a large NHANES sample, standardized assessment of depressive symptoms, 16S rRNA oral microbiome data processed within a common analytic resource, and sequential multivariable adjustment. Additional analyses included distributional assessment, component-level robustness analyses, multicollinearity diagnostics, leave-one-genus-out recalculation of the oral index, participant-selection comparisons, and exploratory stratification by smoking status and alcohol consumption. The primary indirect association was evaluated using 5000 nonparametric bootstrap resamples.

The limitations are equally important. First, the cross-sectional design precludes conclusions about temporality or causality and leaves open reverse and bidirectional explanations. Second, the Integrated Lifestyle Score was derived within the analytic sample using PHQ-9-related weights and has not been independently validated; it captures only physical activity and dietary inflammatory potential and omits other lifestyle domains, particularly sleep. Third, the Oral Dysbiosis Index is investigator-defined, based on six genera measured at one time point, and has not been validated in an independent cohort. Fourth, complete-case selection may introduce selection bias. Included participants were younger and had slightly higher BMI than nonincluded adults, and the final sample was restricted to ages 20–69 years. Fifth, the 2009–2012 NHANES cycles are relatively old. They were used because they provided the required oral microbiome, dietary, physical-activity, and depressive-symptom measures concurrently, but changes in diet, behavior, oral health, and population characteristics may limit direct generalization to contemporary U.S. adults. Sixth, important oral-health confounders, including detailed oral-hygiene practices, periodontal status, dental treatment, and relevant medication exposures, were not comprehensively represented. Finally, the primary bootstrap used individual-level case resampling rather than a fully design-based replicate-weight bootstrap, so the indirect-association estimates should be regarded as exploratory.

## 5. Conclusions

In this survey-weighted analysis of U.S. adults, a less favorable profile characterized by higher dietary inflammatory potential and lower leisure-time physical activity was associated with greater depressive symptom burden and a higher study-specific Oral Dysbiosis Index. Oral dysbiosis accounted statistically for a small proportion of the lifestyle–depressive symptom association. These findings support further longitudinal and interventional investigation of lifestyle, oral microbial ecology, and mental health, but the cross-sectional design and study-specific indices preclude causal interpretation.

## Figures and Tables

**Table 1:** Participant Characteristics According to Lifestyle Profile.

Characteristic	Total (N = 6519)	More Favorable Lifestyle (*n* = 3260)	Less Favorable Lifestyle (*n* = 3259)	*p*-Value
Age (years), mean ± SD	43.2 ± 14.0	43.2 ± 14.0	43.3 ± 14.1	0.86
Female, %	50%	41%	62%	<0.001 *
BMI (kg/m^2^), median (IQR)	27.6 (24.1, 32.4)	26.9 (23.8, 31.2)	28.7 (24.6, 33.7)	<0.001 *
Race/Ethnicity, %				<0.001 *
- Non-Hispanic White	67%	71%	63%	
- Non-Hispanic Black	12%	9%	15%	
- Hispanic	14%	13%	15%	
- Other	7%	7%	6%	
Education Level, %				<0.001 *
- Less than high school	15%	10%	20%	
- High school	20%	17%	24%	
- College or above	65%	72%	56%	
Household Income (PIR), %				<0.001 *
- Low (<1)	17%	14%	21%	
- Middle (1–3)	33%	28%	38%	
- High (≥3)	50%	58%	41%	
Marital Status, %				0.094
- Married/Living with partner	61%	63%	59%	
- Widowed/Divorced/Separated	16%	14%	18%	
- Never married	23%	23%	23%	
Smoking Status, %				<0.001 *
- Never	55%	58%	52%	
- Former	22%	24%	20%	
- Current	22%	17%	29%	
Alcohol Use, %				<0.001 *
- None	71%	66%	78%	
- Moderate	13%	16%	10%	
- High	16%	18%	12%	
BMI Category, %				<0.001 *
- <25	31%	34%	27%	
- 25–<30	32%	35%	30%	
- ≥30	36%	31%	43%	
DII, mean ± SD	0.7 ± 1.8	−0.5 ± 1.5	2.3 ± 0.9	<0.001 *
Physical Activity (MET-min/week), median (IQR)	360.0 (0.0, 1320.0)	960.0 (0.0, 2160.0)	0.0 (0.0, 480.0)	<0.001 *
Integrated Lifestyle Score, median (IQR)	0.0 (−0.5, 0.5)	−0.5 (−0.9, −0.2)	0.5 (0.3, 0.8)	<0.001 *
Oral Dysbiosis Index, median (IQR)	1.4 (−0.1, 3.5)	1.1 (−0.3, 3.1)	1.8 (0.1, 4.0)	<0.001 *
PHQ-9 Score, median (IQR)	2.0 (0.0, 4.0)	1.0 (0.0, 4.0)	2.0 (0.0, 5.0)	<0.001 *
Clinically Relevant Depressive Symptoms (PHQ-9 ≥ 10), %	9%	7%	11%	<0.001 *

Note: Values are survey-weighted unless otherwise indicated. Approximately symmetric continuous variables are presented as mean ± SD; skewed continuous variables as median (Q1, Q3); and categorical variables as weighted percentages. Group comparisons used survey-weighted t tests for approximately symmetric variables, survey-weighted rank tests for skewed variables, and Rao–Scott adjusted chi-square tests for categorical variables. Participants were divided at the sample median of the Integrated Lifestyle Score (0.112) for descriptive comparisons only. Higher scores indicate less favorable profiles characterized by higher dietary inflammatory potential and lower physical activity. * *p* < 0.05 (two-sided). Abbreviations: BMI, body mass index; DII, Dietary Inflammatory Index; IQR, interquartile range; MET, metabolic equivalent task; PHQ-9, Patient Health Questionnaire-9; PIR, poverty–income ratio; SD, standard deviation.

**Table 2:** Association Between Integrated Lifestyle Score and Depressive Symptoms.

Model	OR	Lower 95% CI	Upper 95% CI	*p*-Value
Model 1 (Crude)	2.006	1.698	2.371	<0.001 *
Model 2 (Sociodemographic-adjusted)	1.565	1.326	1.847	<0.001 *
Model 3 (Fully adjusted)	1.355	1.159	1.584	<0.001 *
Outcome: PHQ-9 Score (continuous)				
**Model**	**β**	**SE**	**95% CI**	***p*** **-Value**
Model 1 (Crude)	0.836	0.096	0.641–1.031	<0.001 *
Model 2 (Sociodemographic-adjusted)	0.514	0.097	0.312–0.716	<0.001 *
Model 3 (Fully adjusted)	0.314	0.094	0.113–0.515	0.005 *

Note: The upper panel reports clinically relevant depressive symptoms (PHQ-9 ≥ 10). Model 1 was unadjusted. Model 2 adjusted for age, sex, race/ethnicity, educational attainment, PIR, and marital status. Model 3 additionally adjusted for BMI, smoking status, and alcohol consumption. * *p* < 0.05 (two-sided). Abbreviations: BMI, body mass index; CI, confidence interval; OR, odds ratio; PHQ-9, Patient Health Questionnaire-9; PIR, poverty–income ratio; SE, standard error.

**Table 3:** Association Between Integrated Lifestyle Score and Oral Dysbiosis Index.

Model	β	SE	Lower 95% CI	Upper 95% CI	*p*-Value
Model 1 (Crude)	0.442	0.080	0.278	0.606	<0.001 *
Model 2 (Sociodemographic-adjusted)	0.334	0.074	0.180	0.488	<0.001 *
Model 3 (Fully adjusted)	0.191	0.066	0.050	0.331	0.011 *

Note: The Oral Dysbiosis Index was analyzed as a continuous, study-specific ecological log-ratio measure. Model definitions are as in Table 2. * *p* < 0.05 (two-sided). Abbreviations: CI, confidence interval; SE, standard error.

**Table 4:** Association Between Oral Dysbiosis Index and Depressive Symptoms.

Model	OR	Lower 95% CI	Upper 95% CI	*p*-Value
Model 1 (Crude)	1.110	1.081	1.139	<0.001 *
Model 2 (Sociodemographic-adjusted)	1.096	1.065	1.129	<0.001 *
Model 3 (Fully adjusted)	1.069	1.035	1.105	<0.001 *
Outcome: PHQ-9 Score (continuous)				
**Model**	**β**	**SE**	**95% CI**	***p*** **-Value**
Model 1 (Crude)	0.169	0.020	0.127–0.210	<0.001 *
Model 2 (Sociodemographic-adjusted)	0.138	0.019	0.099–0.178	<0.001 *
Model 3 (Fully adjusted)	0.101	0.020	0.059–0.144	<0.001 *

Note: The upper panel reports clinically relevant depressive symptoms (PHQ-9 ≥ 10). Model definitions are as in Table 2. * *p* < 0.05 (two-sided). Abbreviations: CI, confidence interval; OR, odds ratio; PHQ-9, Patient Health Questionnaire-9; SE, standard error.

## Data Availability

The data analyzed in this study are publicly available from the National Health and Nutrition Examination Survey (NHANES), conducted by the Centers for Disease Control and Prevention, and can be accessed at https://www.cdc.gov/nchs/nhanes/ (accessed on 15 August 2026).

## Supplementary Materials

The following supporting information can be downloaded at https://www.mdpi.com/article/10.3390/healthcare14172795/s1: Supplementary Methods; Table S1: Sample Characteristics and Distributional Assessment; Table S2: Component-Level Robustness Analyses of Physical Activity and Dietary Inflammatory Potential; Table S3: Model and Oral Microbial Index Robustness Analyses; Table S4: Exploratory Stratified Analyses by Smoking Status and Alcohol Consumption for Continuous PHQ-9.

## Abbreviations

BMI, body mass index; CI, confidence interval; DII, Dietary Inflammatory Index; GVIF, generalized variance inflation factor; MET, metabolic equivalent task; NHANES, National Health and Nutrition Examination Survey; OR, odds ratio; PHQ-9, Patient Health Questionnaire-9; PIR, poverty–income ratio; PSU, primary sampling unit; SE, standard error; VIF, variance inflation factor.
