# Supplementary material for: Physical Activity, Dietary Inflammatory Potential, and Depressive Symptoms Among U.S. Adults: The Potential Contribution of Oral Microbial Dysbiosis
Source: Healthcare (Basel). 2026 Sep 1;14(17):2795. doi: 10.3390/healthcare14172795 (PMC13564869; doi:10.3390/healthcare14172795)
Supplement: Supplementary file 1 [file healthcare-14-02795-s001.zip › healthcare-4478524-supplementary.pdf]

Supplementary Materials

Physical Activity, Dietary Inflammatory Potential, and Depressive Symptoms among U.S. Adults: The Potential Contribution of Oral Microbial Dysbiosis

Supplementary Methods

The Dietary Inflammatory Index was calculated from 26 parameters available in the Day 1 NHANES dietary recall files: energy, protein, carbohydrate, fiber, total fat, saturated fat, monounsaturated fat, polyunsaturated fat, cholesterol, iron, magnesium, zinc, selenium, vitamin A, thiamin, riboflavin, niacin, vitamin B6, vitamin B12, folate, vitamin C, vitamin D, vitamin E, β-carotene, caffeine, and alcohol.

Supplementary Table S1. Sample Characteristics and Distributional Assessment

Panel A. Comparison of included and nonincluded adult participants

Characteristic	Included (n=6519)	Nonincluded (n=5872)	P value
Age (years), mean ± SD	43.6 ± 14.4	52.4 ± 21.6	<0.001
Female, %	50.3%	51.8%	0.091
Non-Hispanic White, %	41.0%	42.8%	0.054
BMI (kg/m²), mean ± SD	29.3 ± 7.1	28.3 ± 6.6	<0.001

Note: This comparison was unweighted and was used only to characterize potential selection differences between the final analytic sample and other adults aged ≥18 years in NHANES 2009–2012.

Panel B. Distributional characteristics of continuous variables in the analytic sample

Variable	Mean	SD	Median	Q1	Q3	Minimum	Maximum	Skewness
Age (years)	43.64	14.42	43.00	31.00	56.00	20.00	69.00	0.03
BMI (kg/m²)	29.29	7.07	28.10	24.30	32.85	13.60	82.10	1.27
Physical activity (MET-min/week)	854.90	1602.75	160.00	0.00	1080.00	0.00	21600.00	3.94
Dietary Inflammatory Index	0.97	1.79	1.17	−0.31	2.43	−4.94	4.84	−0.44
Oral Dysbiosis Index	1.99	3.47	1.40	−0.10	3.53	−19.37	19.37	1.16
PHQ-9 score	3.40	4.52	2.00	0.00	5.00	0.00	27.00	1.97

Note: These unweighted diagnostics were used to characterize distributional shape. In the main Table 1, age and DII were summarized as survey-weighted mean ± SD, whereas BMI, physical activity, the Integrated Lifestyle Score, Oral Dysbiosis Index, and PHQ-9 were summarized as survey-weighted median (Q1, Q3) because of evident skewness. After recalculation with the final WTDRD1-based lifestyle weights, the Integrated Lifestyle Score ranged from −5.76 to 1.40 and had a sample median of 0.112; its survey-weighted descriptive distribution is reported in the main Table 1.

Supplementary Table S2. Component-Level Robustness Analyses of Physical Activity and Dietary Inflammatory Potential

Panel A. Continuous PHQ-9 score

Exposure	Model	β	Lower 95% CI	Upper 95% CI	P value
Physical activity, Z(PA)	Crude	−0.366	−0.511	−0.220	<0.001
Physical activity, Z(PA)	Model 2	−0.267	−0.410	−0.124	<0.001
Physical activity, Z(PA)	Model 3	−0.160	−0.304	−0.017	0.031
Physical activity, Z(PA)	Model 3 (joint)	−0.147	−0.293	−0.002	0.047
Dietary inflammation, Z(DII)	Crude	0.574	0.445	0.702	<0.001
Dietary inflammation, Z(DII)	Model 2	0.301	0.151	0.452	<0.001
Dietary inflammation, Z(DII)	Model 3	0.178	0.024	0.333	0.026
Dietary inflammation, Z(DII)	Model 3 (joint)	0.166	0.006	0.326	0.042

Panel B. Clinically relevant depressive symptoms (PHQ-9 ≥10)

Exposure	Model	OR	Lower 95% CI	Upper 95% CI	P value
Physical activity, Z(PA)	Crude	0.702	0.560	0.881	0.003
Physical activity, Z(PA)	Model 2	0.798	0.666	0.956	0.017
Physical activity, Z(PA)	Model 3	0.860	0.729	1.015	0.071
Physical activity, Z(PA)	Model 3 (joint)	0.873	0.736	1.034	0.108
Dietary inflammation, Z(DII)	Crude	1.531	1.392	1.684	<0.001
Dietary inflammation, Z(DII)	Model 2	1.302	1.151	1.472	<0.001
Dietary inflammation, Z(DII)	Model 3	1.192	1.045	1.359	0.012
Dietary inflammation, Z(DII)	Model 3 (joint)	1.180	1.028	1.355	0.021

Panel C. Associations of individual lifestyle components with the Oral Dysbiosis Index

Exposure	Model	β	SE	Lower 95% CI	Upper 95% CI	P value
Physical activity (per 1000 MET-min/week)	Crude	−0.124	0.022	−0.168	−0.079	<0.001
Physical activity (per 1000 MET-min/week)	Model 2	−0.085	0.018	−0.123	−0.048	<0.001
Physical activity (per 1000 MET-min/week)	Model 3	−0.040	0.018	−0.076	−0.004	0.031
Dietary inflammatory index (per 1 unit)	Crude	0.166	0.036	0.088	0.244	<0.001
Dietary inflammatory index (per 1 unit)	Model 2	0.127	0.036	0.048	0.206	0.003
Dietary inflammatory index (per 1 unit)	Model 3	0.077	0.034	0.004	0.150	0.039

Note: Model 2 adjusted for age, sex, race/ethnicity, educational attainment, poverty-income ratio, and marital status. Model 3 additionally adjusted for BMI, smoking status, and alcohol consumption. In Panels A and B, joint models included Z(PA) and Z(DII) simultaneously. These analyses were conducted as robustness analyses and do not constitute validation of the Integrated Lifestyle Score.

Supplementary Table S3. Model and Oral Microbial Index Robustness Analyses

Panel A. Multicollinearity diagnostics for the fully adjusted model

Variable	df	VIF-like value
Integrated Lifestyle Score	1	1.168
Age	1	1.404
Sex	1	1.134
Race/Ethnicity	3	1.093
Education	2	1.176
Income (PIR)	2	1.158
Marital status	2	1.191
BMI category	2	1.064
Smoking status	2	1.121
Alcohol use	2	1.053

Note: For multi-level categorical variables, generalized variance inflation factors were adjusted for degrees of freedom and converted to a VIF-like value for comparison with the conventional threshold of 5. The maximum VIF-like value was 1.40.

Panel B. Leave-one-genus-out analysis of the study-specific Oral Dysbiosis Index

Genus removed	Index component	β (PHQ-9)	SE	P value	Correlation with full index
None (full six-genus index)	—	0.101	0.020	<0.001	1.000
Lactobacillus	Numerator	0.101	0.021	<0.001	0.986
Scardovia	Numerator	0.100	0.020	<0.001	0.996
Veillonella	Numerator	0.047	0.011	<0.001	0.702
Neisseria	Denominator	0.090	0.022	0.001	0.814
Aggregatibacter	Denominator	0.102	0.019	<0.001	0.979
Capnocytophaga	Denominator	0.077	0.016	<0.001	0.917

Note: Each analysis recalculated the index after removing one genus and then re-estimated its association with continuous PHQ-9 in the fully adjusted model. The consistently positive estimates support internal robustness but do not constitute external validation.

Supplementary Table S4. Exploratory Stratified Analyses by Smoking Status and Alcohol Consumption for Continuous PHQ-9

Panel A. Smoking-status strata

Stratum	N	Path a	Path b	Indirect effect
Never	3643	0.109	0.104	0.0113
Former	1343	0.326	0.118	0.0385
Current	1533	0.251	0.087	0.0218

Panel B. Alcohol-consumption strata

Stratum	N	Path a	Path b	Indirect effect
None	4820	0.261	0.106	0.0278
Moderate	757	0.253	0.219	0.0554
High	942	0.165	−0.013	−0.0021

Note: These subgroup analyses were exploratory and are presented as descriptive point estimates only. Within smoking-status strata, smoking status was omitted from the adjustment set; within alcohol-consumption strata, alcohol consumption was omitted. No subgroup-specific bootstrap inference or formal between-stratum comparisons were performed. The primary overall indirect-association analysis used 5,000 nonparametric bootstrap resamples.

Abbreviations: BMI, body mass index; CI, confidence interval; DII, Dietary Inflammatory Index; MET, metabolic equivalent task; OR, odds ratio; PHQ-9, Patient Health Questionnaire-9; PIR, poverty-income ratio; SD, standard deviation; SE, standard error; VIF, variance inflation factor.
